# Nebulised amphotericin B to eradicate Candida colonisation from the respiratory tract in critically ill patients receiving selective digestive decontamination: a cohort study

**DOI:** 10.1186/cc13056

**Published:** 2013-10-11

**Authors:** David SY Ong, Peter MC Klein Klouwenberg, Cristian Spitoni, Marc JM Bonten, Olaf L Cremer

**Affiliations:** 1 Department of Intensive Care Medicine, University Medical Center Utrecht, Heidelberglaan 100, 3584, CX Utrecht, The Netherlands; 2 Department of Medical Microbiology, University Medical Center Utrecht, Heidelberglaan 100, 3584, CX Utrecht, The Netherlands; 3 Julius Center for Health Sciences and Primary Care, University Medical Center Utrecht, Heidelberglaan 100, 3584, CX Utrecht, The Netherlands; 4 Department of Mathematics, Utrecht University, Budapestlaan 6, 3584, CD Utrecht, The Netherlands

## Abstract

**Introduction:**

Colonisation of the lower respiratory tract with *Candida* species occurs in 25% of mechanically ventilated critically ill patients, and is associated with increased morbidity. Nebulised amphotericin B has been used to eradicate *Candida* as part of selective decontamination of the digestive tract (SDD) protocols, but its effectiveness is unknown. We aimed to determine the effectiveness of nebulised amphotericin B in eradicating *Candida* respiratory tract colonisation in patients receiving SDD.

**Methods:**

We included consecutive mechanically ventilated patients during a four-year period. Microbiological screening was performed upon admission and twice weekly thereafter according to a standardised protocol. A colonisation episode was defined as the presence of *Candida* species in two consecutive sputum samples taken at least one day apart. To correct for time-varying bias and possible confounding, we used a multistate approach and performed time-varying Cox regression with adjustment for age, disease severity, *Candida* load at baseline and concurrent corticosteroid use.

**Results:**

Among 1,819 patients, colonisation with *Candida* occurred 401 times in 363 patients; 333 of these events were included for analysis. Decolonisation occurred in 51 of 59 episodes (86%) and in 170 of 274 episodes (62%) in patients receiving and not receiving nebulised amphotericin B, respectively. Nebulised amphotericin B was associated with an increased rate of *Candida* eradication (crude HR 2.0; 95% CI 1.4 to 2.7, adjusted HR 2.2; 95% CI 1.6 to 3.0). Median times to decolonisation were six and nine days, respectively. The incidence rate of ventilator-associated pneumonia, length of stay and mortality did not differ between both groups.

**Conclusions:**

Nebulised amphotericin B reduces the duration of *Candida* colonisation in the lower respiratory tracts of mechanically ventilated critically ill patients receiving SDD, but data remain lacking that this is associated with a meaningful improvement in clinical outcomes. Until more evidence becomes available, nebulised amphotericin B should not be used routinely as part of the SDD protocol.

## Introduction

*Candida* species are opportunistic pathogens that ordinarily inhabit the human gastrointestinal tract. Colonisation of the lower respiratory tract (LRT) by *Candida* occurs in 25% of critically ill patients receiving mechanical ventilation and in 50% of patients suspected of ventilator-associated pneumonia (VAP), and has been associated with longer intensive care unit (ICU) stay, a prolonged duration of mechanical ventilation, an increased risk of bacterial VAP, and possibly increased in-hospital mortality [1-4]. Whether the presence of *Candida* is a cause or merely a marker of a more severe clinical course is uncertain.

Furthermore, it remains unclear how to differentiate between colonisation and infection of the LRT. In general, *Candida* species are not assumed to be primary causative pathogens in VAP patients [5]. In a post-mortem study in patients with evidence of pneumonia at autopsy, none of the subjects with a tracheal aspirate or bronchoalveolar lavage culture positive for *Candida* species had histopathological evidence of invasive *Candida* growth [6]. However, there is evidence that colonisation of the LRT by *Candida* species promotes the development of pneumonia by creating biofilms that are capable of holding other micro-organisms [7]. Moreover, *Candida* is assumed to have an indirect effect by decreasing the immune defence and favouring bacterial development [8]. Experimental and clinical studies have shown that *Candida* colonisation was associated with an increased risk for VAP by *Pseudomonas aeruginosa*, and that systemic antifungal treatment decreased the risk for *P*. *aeruginosa* infection in colonised patients [1,9,10].

Side effects associated with systemic antifungal treatment have limited the use of pre-emptive strategies for *Candida* colonisation to patients who are at high-risk for invasive candidiasis, such as patients with severe and multiple-site colonisation [11]. However, local use of antifungal medication may provide a potentially attractive alternative approach. Nebulised amphotericin B (NAB) has been used to eradicate *Candida* species from the LRT as part of various selective decontamination of the digestive tract (SDD) protocols in patients with persistent *Candida* colonisation despite topical use of amphotericin B [12]. However, the clinical effectiveness of this approach in reducing the burden of *Candida* colonisation in ICU patients is not known.

## Materials and methods

### Patients and measurements

This study was performed in a 32-bed mixed ICU of the University Medical Center Utrecht in the Netherlands between April 2008 and February 2012. The Ethics Committee of the University Medical Center Utrecht approved this study and waived the need for informed consent. We analysed all mechanically ventilated adults who had been admitted to the ICU for >72 hours. Microbiological screening for the presence of *Candida* was prospectively carried out on admission and twice weekly thereafter according to a standardised protocol that was part of a SDD/Selective Oropharyngeal Decontamination (SOD) trial [12]. In brief, samples were inoculated on malt extract agar plates, and *Candida* load was semi-quantitatively determined (that is classified as <10, 10 to 100, and >100 colony-forming units). Colonisation was defined as the presence of *Candida* species in two or more consecutive bronchoalveolar lavage or sputum samples obtained on different days in the ICU, and the colonisation start date as the first positive sample. Decolonisation was defined as the absence of *Candida* in two consecutive samples on different days, or as the absence of *Candida* in the last available sample. We excluded episodes during which patients had received systemic antifungal treatment, as well as episodes during which NAB was initiated within the first two days after colonisation start date (before results of microbiological surveillance cultures had become available), because the reason for use of amphotericin B in these cases was likely to be different.

### Decontamination protocol

All patients received either SDD (from April 2008 to August 2009 and from June 2011 to February 2012) or SOD (from September 2009 to May 2011). During SDD (but not during SOD) NAB, four times 5 mg daily, was recommended in case of *Candida* colonisation of the LRT [12]. To this end, 50 mg of conventional amphotericin B for intravenous use was dissolved in 10 mL water for injection. For each nebulisation session 2 mL of water for injection was added to 1 mL (= 5 mg amphotericin B) of the prepared solution and aerosolised with a jet nebuliser system (Covidien, Mansfield, MA, USA). Air or oxygen under high pressure with a flow rate of 5 to 8 L per minute was used to generate aerosols with a mass median aerodynamic diameter of <5 micrometer. Each session lasted until all medication was inhaled (about 15 to 30 minutes). The nebuliser was attached to the ventilator circuit with a T-piece adaptor positioned between the endotracheal tube and the humidification filter. The nebulisation protocol did not recommend specific ventilator settings. In our clinical practice, however, patients remained on a pressure-controlled ventilator with unchanged positive end-expiratory pressure (PEEP) and inspiratory pressure settings. Depending on the mechanical ventilator type nebulisation was continuously administered during both inspiration and expiration in half of the NAB treatments, whereas in the remaining cases nebulisation was only during inspiration. Allocation of a ventilator depended on availability and was independent of patient characteristics. The humidification filter in the mechanical ventilation circuit was changed after each nebulisation session to avoid obstruction.

### Data analysis

Because the delay in the initiation of NAB treatment (relative to the start of colonisation) may vary between patients, it is important to correct for time-varying bias, which occurs when the exposure variable is categorised to its final status rather than considering the timing of the change in status [13,14]. Furthermore, patients who spontaneously decolonise early have less opportunity to receive NAB. These cases contribute better outcomes to the non-exposed group and therefore may lead to an underestimation of the treatment effect. To deal with these issues, we used time-varying Cox proportional hazards regression with adjustment for age, Sequential Organ Failure Assessment (SOFA) score, *Candida* load at baseline, and concurrent use of corticosteroids.

**Figure 1:**
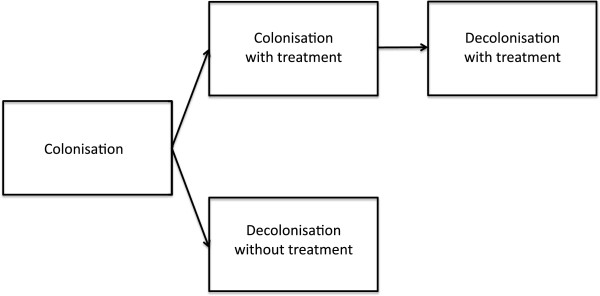
**Multistate model.** This multistate model was used for estimating the median time to decolonisation relative to the start of nebulised amphotericin B, and for graphically representing colonisation persistence probability curves in Figure 4. A patient with colonisation remained in this state as long as no treatment was given. Patients transitioned to the 'colonisation with treatment’ state upon receiving treatment, and subsequently transitioned to 'decolonisation with treatment’ state when decolonisation occurred. The patient underwent transition to 'decolonisation without treatment’ state in case decolonisation occurred without treatment or before treatment was started. Thus, two states were transitional states, whereas the remaining two states were final states in our model (meaning that no further data were included in the model beyond this point in time).

In order to graphically represent the results of our time-varying analysis and correctly estimate the median time to decolonisation relative to the start of NAB [15], we used the multistate model shown in Figure 1[16].

As a secondary study outcome, we compared the incidence rates of VAP occurring after the onset of the *Candida* colonisation episode in both groups. For the period 2008 to 2010, we retrospectively assessed the medical records for the occurrence of VAP, according to established CDC criteria [17]. For the period 2011 to 2012, we prospectively recorded the incidences of VAP as part of an ongoing cohort study that is aimed at finding early diagnostic and prognostic markers of sepsis [18]. Furthermore, we compared ICU mortality and remaining length of stay in ICU following the onset of *Candida* colonisation in patients receiving NAB compared to patients not receiving NAB.

To assess possible effect modification by the timing of treatment, we performed a secondary analysis on the overall treatment effect by comparing patients who had received NAB treatment early (within five days) versus late (after five days) following the colonisation start date. Because of a reduced number of decolonisation events in this subgroup analysis of early and late NAB starters, we tested possible confounders by adding each of them separately to a univariable Cox regression model containing only NAB as an explanatory variable and examining its effect on the beta coefficient of the NAB variable on decolonisation. Covariables that caused substantial confounding (that is a change in effect estimate greater than 10%) were included in the final multivariable models. In addition, to assess possible indication bias we performed a per-protocol analysis of patients treated according to the SDD and SOD arms of the trial. Data were analysed with SAS 9.2 (SAS Institute, Inc., Cary, NC, USA) and R 2.15.1 software (package mstate).

## Results

**Figure 2:**
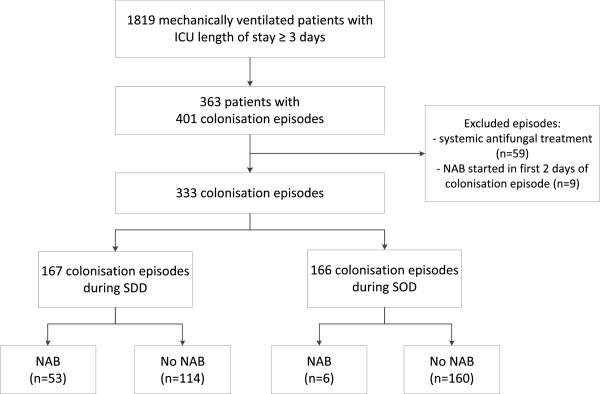
Patient inclusion.

Out of a total of 1,819 patients who had been admitted to the ICU for at least 72 hours and received mechanical ventilation, colonisation with *Candida* occurred 401 times in 363 patients (Figure 2). Colonisation rates did not differ between the SDD and SOD periods (21% versus 19% respectively, *P* = 0.44). After exclusion of colonisation episodes in which concurrent systemic antifungal treatment was administered (n = 59) and episodes in which NAB were started within the first two days after the start of colonisation (n = 9), 333 episodes remained for analysis.

Almost half of the patients (48%) were already colonised upon ICU admission. These patients did not have significantly more colonisation episodes compared to patients who acquired colonisation during the ICU admission (*P* = 0.09). Overall, 44% of positive cultures were classified as *Candida albicans*, 11% as *Candida glabrata*, and 8% as *non*-*albicans/non-glabrata/non-krusei* species (37% of cultures were not further subtyped). Concurrent oropharyngeal *Candida* co-colonisation was present in 247 out of the 317 (78%) initial colonisation episodes, and in 10 out of the 16 (63%) recolonisation episodes.

**Figure 3:**
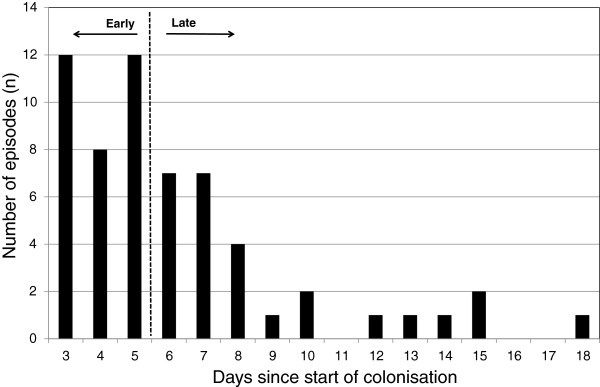
**Time delay between start of *Candida *colonisation and treatment initiation.** This figure depicts the observed time delay between the start of the *Candida* colonisation episode and the initiation of nebulised amphotericin B treatment. Episodes during which treatment was initiated within the first two days after start of colonisation (before results of microbiological surveillance cultures had become available) were excluded from the study, because the reason for antifungal treatment in these cases is likely to be different.

The initial *Candida* load in the sputum was significantly higher in patients receiving amphotericin B compared to those not receiving therapy (Table 1). However, the number of bacterial co-colonisations was similar in both groups at baseline. There was also considerable variation in the time delay between the start of *Candida* colonisation and start of therapy, with 46% of patients starting treatment after more than five days (Figure 3).

**Table 1:** Baseline characteristics

***Characteristics***	***Nebulised amphotericin B (n = 59)***	***Standard care (n = 274)***	***P value***
Age	63 (43–76)	63 (52–72)	0.78
Gender male	43 (73)	189 (69)	0.55
APACHE IV score	77 (60–96)	75 (58–92)	0.42
Corticosteroid use^a^	18 (31)	75 (27)	0.63
SOFA score^b^	6 (3–8)	5 (3–8)	0.51
Days in ICU before onset of the colonisation episode	0 (0–2)	1 (0–3)	0.24
*Candida* load in sputum (CFUs)^c^			<0.001
*<10*	9 (15)	117 (43)	
*10-100*	32 (54)	102 (37)	
*>100*	18 (31)	55 (20)	
*Candida* colonisation of the oropharynx	50 (85)	213 (78)	0.23
*Candida* colonisation of the rectum	23 (39)	75 (27)	0.08
Delay between onset of *Candida* colonisation in sputum and NAB start	5 (4–7)	NA	NA
Bacterial co-colonisation in sputum:			
Gram-negative rods^d^	13 (22)	83 (30)	0.20
Pseudomonas species	1 (2)	20 (7)	0.11
Gram-positive cocci	13 (22)	63 (23)	0.87

^a^Corticosteroid use was defined as a daily dose >100 mg hydrocortisone or equivalent; ^b^we used a modified sum score, excluding points for the central nervous system; ^c^*Candida* load at baseline was determined by semi-quantitative culture; ^d^Gram-negative rods including *Pseudomonas* species. APACHE, Acute Physiology and Chronic Health Evaluation; CFU, colony-forming units; ICU, intensive care unit; NA, not applicable; SDD, selective digestive decontamination; SOD, selective oral decontamination; SOFA, Sequential Organ Failure Assessment. Data are presented as medians (interquartile range (IQR)) or absolute numbers (%).

Decolonisation occurred in 51 of 59 (86%) and 170 of 274 (62%) episodes in patients receiving and not receiving NAB, respectively. Following the start of NAB treatment the proportion of sputum cultures growing *Candida* decreased over time (Table 2), suggesting true decolonisation rather than *in vitro* suppression of growth by amphotericin. Recolonisation occurred in 2% and 6% of patients receiving and not receiving NAB, respectively (Table 3).

**Table 2:** **Proportion of consecutive sputum cultures showing *Candida *** **growth following the start of nebulised amphotericin B (NAB) treatment**

**Consecutive sputum cultures following the start of NAB**	**Proportion of patients with positive culture (%)**
1	100
2	67
3	25
4	19
5	9

**Table 3:** Univariable analysis of secondary outcomes

***Characteristics***	***NAB (n = 59)***	***Standard care (n = 274)***	***P value***
VAP incidence after the onset of *Candida* colonisation (per 1000 ICU days)	6.5	5.5	0.64
Number of *Candida* recolonisation episodes*	1 (2)	15 (6)	0.32
Length of stay in ICU after onset of *Candida* colonisation	23 (12–30)	14 (10–23)	0.004
Length of stay in ICU after NAB start	14 (7–25)	NA	NA
ICU Mortality	10 (17)	54 (20)	0.62

^*^The denominators for the calculation of the numbers of *Candida* recolonisation events in the NAB and standard care group are 58 and 259 initial colonisation episodes, respectively. ICU, intensive care unit; NA, not applicable; NAB, nebulised amphotericin B; VAP, ventilator-associated pneumonia. Data are presented as medians (interquartile range (IQR)) or absolute numbers (%), except for the ventilator-associated pneumonia rate that is presented as the number per 1,000 ICU days.

**Figure 4:**
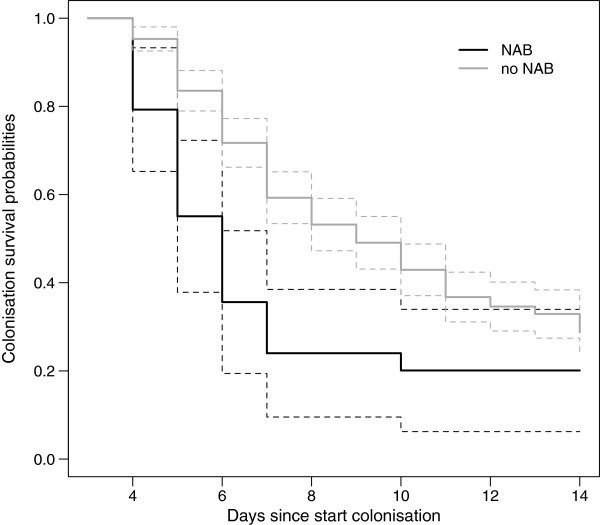
**Time to *Candida *eradication of the lower respiratory tract.** Colonisation persistence probability curves of the nebulised amphotericin B (NAB) group and the no NAB group were plotted with 95% confidence intervals using the multistate approach. Both curves were conditioned on being colonised at day 4 in order to have a meaningful comparison between the treated and untreated patients. On any given day, one curve represented episodes in which NAB treatment was given and the other represented those without treatment.

Figure 4 shows the persistence probability of colonisation as analysed using a multistate approach. Both curves are conditioned on colonisation being present until at least day 4. Median times to elimination of *Candida* were estimated as six (interquartile range (IQR) 5 to 7) days and nine (IQR 6 to 20) days in patients receiving and not receiving NAB, respectively. In time-varying analyses, NAB treatment was associated with an approximately two-fold increase in the rate of decolonisation (crude hazard ratio (HR) 2.0; 95% confidence interval (CI) 1.4 to 2.7, adjusted HR 2.2; 95% CI 1.6 to 3.0).

The incidence rate of VAP and ICU mortality did not differ between both groups (Table 3). The length of stay in ICU after NAB initiation was comparable to the length of stay in ICU after onset of *Candida* colonisation in the control group, 14 (IQR 7 to 25) and 14 (10 to 23) days, respectively. In 5 of 59 episodes NAB treatment was discontinued before successful decolonisation was achieved, because of extubation (n = 2), clinical decision to stop all antimicrobial therapy (n = 1) or unclear reasons (n = 2). No adverse effects (such as cough or bronchospasm) were documented in the medical records for any of the 59 cases.

### Secondary analysis

There were 27/31 (87%) and 24/28 (86%) decolonisation events in early and late NAB starters, respectively, resulting in an adjusted HR of 2.0 (95% CI 1.3 to 3.1), and 2.0 (95% CI 1.3 to 3.2), respectively. During the SOD period, in which NAB treatment was not recommended in the protocol, NAB was applied in 6 of 166 (4%) cases, whereas during the SDD period 114 of 167 (68%) cases did not receive NAB while being colonised. However, after exclusion of episodes during SDD in which NAB was not started as well as episodes during SOD in which NAB was administrated (per-protocol analysis), the effect estimates remained similar (crude HR 2.1; 95% CI 1.4 to 3.0, adjusted HR 2.2; 95% CI 1.5 to 3.3).

## Discussion

Inhalation therapy with NAB resulted in a three-day reduction in the duration of *Candida* colonisation in the LRT of mechanically ventilated patients who are (also) receiving topical applications of amphotericin B as part of a SDD protocol. This effect remained after adjustment for potential confounders and was approximately constant irrespective of the duration of colonisation prior to the start of treatment.

Although NAB has been an essential component in SDD protocols for many years [12,19], this is the first study to assess the effectiveness of inhalation treatment with NAB for eradication of *Candida* in colonised ICU patients. Previous studies have focused on lung transplant recipients in whom NAB was used as prophylactic treatment for, in particular, *Aspergillus* infection [20-22]. Inhalation treatment seems attractive, because high drug concentrations can be reached at the site of interest while reducing nephrotoxicity and drug interactions [23,24]. However, inhalation of amphotericin B has thus far been restricted to off-label use, due to a lack of effectiveness data and standardised methods for nebulised administration. Although inhalation of NAB is generally well tolerated and considered to be safe [25], various reports of adverse effects, including cough, bronchospasm, dyspnea, nausea, and an unpleasant aftertaste, warrant prudency [26-29]. In our study, we observed only a few cases in which NAB treatment was discontinued before successful decolonisation was achieved and we did not find adverse effects documented in the medical records.

Our study has some limitations. First, the study was performed in an SDD/SOD setting exclusively. This included the daily application of a topical paste containing polymyxin E, tobramycin and amphotericin B to prevent colonisation with Gram-negative bacteria and yeasts in the mouth. In addition, SDD (but not SOD) includes the use of a suspension to selectively decontaminate the digestive tract, and the systemic administration of cefotaxime during the first four days of ICU admission [12]. Oropharyngeal decontamination by oral paste might influence *Candida* colonisation in the lower respiratory tract over time by aspiration of topically applied amphotericin in the oropharynx. However, because in both groups the topical application was entirely the same, we assume that the estimation of the efficacy of NAB in combination with oral paste in comparison to oral paste alone is valid within the SDD setting. Both SDD and SOD have been shown to effectively reduce the incidence of VAP [19,30], therefore it is possible that the clinical relevance of reducing the burden of *Candida* colonisation by NAB treatment will differ between settings with and without SDD.

Moreover, during SDD NAB was not started in all patients eligible for this intervention according to protocol recommendations. This probably reflects indication bias. It is plausible that patients with a longer expected ICU stay or greater disease severity were more likely to receive NAB than patients in better health conditions. However, we reduced the possible effects of indication bias by excluding patients who received NAB before microbiological results had become available and by adjusting for potential confounding variables, such as disease severity and *Candida* load. Furthermore, we performed a per-protocol analysis that yielded similar results. Another source of bias may be related to the large variation in the timing of start of treatment that was observed, also known as immortal time bias [31]. Again, by incorporating a time-varying approach into our analysis, we aimed to minimise such bias.

Although we did not find a trend towards a reduced incidence rate of VAP in patients receiving NAB, we stress that the interpretation of this apparent lack of clinical effectiveness requires caution, because our study lacks the statistical power to detect potentially meaningful differences in VAP rates and because of the presence of some concurrent interventions during the use of SDD for which we did not control.

## Conclusions

Inhalation therapy with NAB is associated with a two-fold increase in the rate of *Candida* decolonisation of the LRT in mechanically ventilated patients receiving SDD, resulting in an average reduction of the colonisation time by approximately three days. However, we found no evidence that effective decolonisation translates into a clinically meaningful reduction in VAP rates, ICU length of stay or mortality. The routine use of NAB in current SDD protocols, therefore, cannot be recommended.

### Key messages

•Inhalation therapy with nebulised amphotericin B (NAB) is commonly used to treat colonisation of the lower respiratory tract by *Candida* species as part of SDD/SOD protocols, but its effectiveness has not been studied.

•NAB therapy reduces the duration of *Candida* colonisation of the lower respiratory tract by approximately three days. However, the incidence rate of ventilator-associated pneumonias was not different between the two groups.

•Even without NAB treatment most patients seem to spontaneously decolonise eventually, possibly due the topical application in the oropharynx as part of SDD/SOD.

•Given the current state of evidence, NAB should not be used routinely as part of SDD.

## Abbreviations

CI: Confidence interval; HR: Hazard ratio; ICU: Intensive care unit; IQR: Interquartile range; LRT: lower respiratory tract; NAB: Nebulised amphotericin B; SDD: Selective digestive decontamination; SOD: Selective oral decontamination; VAP: Ventilator-associated pneumonia.

## Competing interests

This research was performed within the framework of CTMM, the Center for Translational Molecular Medicine (http://www.ctmm.nl), project MARS (grant 04I-201). Marc Bonten has received research funding from the Netherlands Organization of Scientific Research (NWO Vici 918.76.611).

## Authors’ contributions

DO, PK, CS, MB and OC substantially contributed to the conception and design of this study. DO, PK and OC acquired the data. DO and CS performed the data analyses. DO, PK, CS, MB and OC were involved in the interpretation of data. DO drafted the manuscript and all authors revised it critically for important intellectual content. All authors read and approved the final manuscript.
